# High-Efficiency Fermentation of Nattokinase by Recombinant *PSP2* Using Oyster Protein Hydrolysate as a Substrate

**DOI:** 10.3390/foods12061252

**Published:** 2023-03-15

**Authors:** Ming Tian, Chen Ning, Siyuan Peng, Deyu Li, Renyi Jin, Yang Zhang, Zhemin Liu, Haijin Mou, Changliang Zhu

**Affiliations:** College of Food Science and Engineering, Ocean University of China, No. 5 Yushan Road, Qingdao 266003, Chinazhuchangliang@ouc.edu.cn (C.Z.)

**Keywords:** oyster protein hydrolysate, nattokinase, recombinant, fermentation, ACE inhibitory activity

## Abstract

In recent years, cardiovascular and cerebrovascular diseases have been the focus of several studies. In this study, oyster protein hydrolysate was produced via enzyme hydrolysis and used as a fermentation substrate to ferment recombinant strain *PSP2* to produce nattokinase. Using the synergism strategy, fermentation products with fibrinolytic and angiotensin I-converting enzyme (ACE) inhibitory activities were obtained and evaluated. The fermentation medium contained 1.0% trypsin, 1.0% oyster protein hydrolysate, 2.0% maltose, and 0.5% sodium chloride, with an initial pH of 7.0. The maximum nattokinase activity was 390.23 ± 10.24 FU/mL after 72 h of fermentation. The flavor of the product was improved, and heavy metals and volatile salt nitrogen were partially removed via fermentation. The ACE inhibitory activity (IC_50_) of the fermentation products was 1.433 mg/mL. This study provides a novel approach for the development of marine functional foods with hypotensive and antithrombotic properties.

## 1. Introduction

Cardiovascular and cerebrovascular diseases are the leading causes of death, accounting for approximately 16 million annual deaths worldwide, according to the World Health Organization. The public health burden of cardiovascular diseases is expected to continue growing as the prevalence of many cardiovascular risk factors increases [1]. The mortality rates of these diseases are continuously increasing, and there is currently a lack of effective strategies for their prevention and control [2].

Nattokinase (EC 3.4.21.62), a basic serine protease produced by *Bacillus subtilis natto* during fermentation, is composed of 275 amino acids with a molecular weight of 27.7 kDa and pI of 8.6. Ser-His-Asp (D32, H64, S221) constitutes its catalytic center. Nattokinase exhibits considerable activity at pH 5.5 to 9.0 [3,4]. Compared to the first-line thrombolytic drugs (urokinase and streprokinase), nattokinase has several advantages, such as safety, low cost, long half-life, and easy oral administration [5]. Therefore, nattokinase has been receiving increasing attention for its use as a thrombolytic agent or healthcare product in other contexts. In addition, nattokinase can directly act on fibrin, catalyze the conversion of blood fibrinogen into active blood fibrinogen lysozyme by dissolving and crosslinking thrombus, increase the synthesis of thrombolytic factors in vivo, enhance the thrombolytic activity in vivo, and greatly improve the efficiency of dissolving fibrin [6]. Nattokinase also has beneficial effects on blood pressure and lipid levels. Therefore, it is a dietary supplement with a high potential in the food industry [7,8,9]. Nattokinase is usually obtained by fermentation. Compared with solid-state fermentation, liquid-state fermentation displays the advantages of simpler extraction, lower cost, and higher yield. However, the low level of nattokinase activity is a bottleneck in its production and application. Natto’s flavor is a result of volatile compounds, and amines are the main cause of its unpleasant smell [10]; therefore, it is necessary to enhance nattokinase activity and reduce its undesirable flavor.

Hypertension is an important risk factor for disease occurrence and mortality in patients with cardiovascular diseases. Many cardiovascular diseases, such as atrial fibrillation and coronary heart disease, are associated with thrombosis [11,12]. Therefore, nattokinase and antihypertensive substances exert synergistic effects in the treatment of patients with thrombosis. Angiotensin I-converting enzyme (ACE) is the first identified dipeptidyl-carboxy oligopeptidase that has the ability to generate the hypertensive peptide angiotensin II and eliminate the vasodilator effects of bradykinin through its degradation. Therefore, it is a key enzyme for controlling high blood pressure [13]. Food-borne bioactive peptides, especially ACE-inhibitory peptides, can inhibit ACE activity and reduce blood pressure. Oysters are one of the four largest cultured shellfish in China, and their extracts have a variety of health benefits, such as decreasing blood pressure and lipid levels, fighting thrombosis, and preventing cardiovascular disease [14]. Wang et al. isolated and characterized a peptide with the sequence “VVYPWTQRF” from oyster protein hydrolysate, and its ACE inhibitory activity was determined with IC_50_ value of 66 μmol/L in vitro [15]. Proteolytic methods involve the preparation of oyster protein hydrolysate using different proteases to hydrolyze oyster proteins [16,17,18]. However, excessive heavy metals are a major issue with aquatic products. Therefore, various studies have focused on reducing the levels of volatile alkaline nitrogen and heavy metals during fermentation.

In this study, we used oyster protein hydrolysate as a fermentation substrate to culture recombinant strain *PSP2*, which is a highly efficient nattokinase producer. Fermentation products with fibrinolytic and ACE inhibitory activities were obtained. The activity of nattokinase in the fermentation liquid was improved by optimizing the fermentation medium and conditions. Heavy metal and total volatile basic nitrogen (TVB-N) levels, flavored substances, and ACE inhibitory activity were also determined.

## 2. Materials and Methods

### 2.1. Bacterial Strain and Culture Conditions

The highly efficient nattokinase-producing *PSP2* strain was obtained using heterologous expression technology with *Bacillus subtilis* as the host. The recombinant strain was maintained at the Applied Microbiology Laboratory, Ocean University of China. A single colony of *B. subtilis PSP2* activated on Luria–Bertani (LB) plates was selected and inoculated into LB medium supplemented with 100 μg/mL kanamycin at 160 rpm and 37 °C for 16 h.

### 2.2. Chemicals

Neutral protease, papain, chitosan, bovine insulin, myoglobin, and tryptophan were purchased from Aladdin Reagent Co., Ltd. (Shanghai, China). Tryptone, yeast extract, Agar, NaCl, glycerin, K_2_HPO_4_·3H_2_O (AR), sorbitol, MgSO_4_·7H_2_O (AR), trisodium citrate, MgCl_2_ (AR), kanamycin, ampicillin, peptone, maltose, sodium acetate, calcium sulfate, acetic acid, Triton X-100, phosphate-buffered saline, normal saline, trichloroacetic acid, and other reagents (AR) were purchased from Sinopharm Chemical Reagent Co., Ltd. (Shanghai, China).

### 2.3. Preparation of Oyster Protein Hydrolysate

Whole oyster meat was homogenized, an appropriate amount of distilled water was added to adjust the solid content to 7.5%, and the pH was adjusted to 7.0. Then, 0.5% neutral protease and 0.25% papain were added to oyster homogenate for enzymolysis at 50 °C for 3 h and inactivated in a boiling water bath for 10 min to obtain the oyster hydrolysate. The pH of the oyster hydrolysate was adjusted to 5.0, and 8.0% chitosan solution with a concentration of 2.0% was added to precipitate the insoluble components and fat in the oyster hydrolysate. After centrifugation, the supernatant was collected and lyophilized at –50 °C to obtain an oyster protein hydrolysate powder.

### 2.4. Enzymatic Activity Assay

The enzymatic activity of nattokinase was determined using UV spectrophotometry, according to the Nattokinase Association of Japan [19]. Fibrin plates were used to verify enzyme production. Fibrinogen level 0.6% (*w*/*v*) in a 50 mM sodium phosphate buffer (pH 7.4) was mixed with 2% (*w*/*v*) agarose and 1 NIH U thrombin. The mixture was poured into a Petri dish and was left for 1 h to form a fibrin clot layer. 20 μL of the enzyme was then dropped on the surface of the fibrin plate, and the plate was incubated at 37 °C for 8–12 h [20].

### 2.5. Best Conditions of Fermentation Conditions

The fermentation conditions were optimized using nattokinase activity as an indicator. The optimization parameters included oyster protein hydrolysate supplementation levels (0, 1, 2, 3, 4, and 5% oyster protein hydrolysate), type of exogenous nitrogen source (soybean peptone, pancreatic peptone, casein peptone, yeast extract, soybean protein powder, and soybean meal), type of carbon source (glucose, xylose, fructose, maltose, and soluble starch), supplemental carbon source (1, 1.5, 2, 2.5, and 3%), initial pH (6, 7, 8, and 9), and fermentation period (0, 24, 48, 72, 96, and 120 h).

### 2.6. ACE Inhibitory Activity

The ACE inhibitory activity of the oyster protein hydrolysate was determined according to the method described by Zhang et al. [14]. First, 20 µL of the sample and 10 µL of ACE solution (0.1 U/mL) were mixed and incubated at 37 °C for 10 min. Thereafter, 10 µL hippuryl–His–Leu (5 mmol/L) solution was added, and the mixture was incubated at 37 °C for 60 min. The reaction was stopped by the addition of 100 µL of 1.0 M HCl. HEPES buffer (20 µL, 50 mmol/mL, pH 8.3) was used as the control instead of the sample. The release of hippuric acid by ACE was measured using a Zorbax SB-Aq C_18_ analytical column via high-performance liquid chromatography(HPLC). The column temperature was 25 °C and the mobile phase was 1:1 acetonitrile:deionized water (containing 0.1% trifluoroacetic acid). The flow rate was 0.3 mL/min and the detection wavelength was 228 nm. The half-maximal inhibitory concentration (IC_50_) was defined as the concentration of the inhibitor that reduced the peak area of hippuric acid by 50%.

ACE inhibition rate  =  (1  −  hippuric acid peak area of sample/hippuric acid peak area of the control group)  ×  100%

### 2.7. Analysis of Fermentation Liquid Components

Solid content analysis was conducted according to the GB 5009.3-2016 National Standard for Food Safety Determination of moisture in food using the direct drying method. Lipid analysis was conducted according to the GB 5009.6-2016 National Standard for Food Safety Determination of fat in food using the Soxhlet extraction method. Total sugar analysis was conducted using the phenol–sulfuric acid method. Total protein analysis was conducted according to the GB 5009.5-2016 National Standard for Food Safety Determination of protein in food for Kjeldahl nitrogen determination. Amino acid composition in liquid samples was determined according to the GB 5009.124-2016 National Standard for Food Safety Determination of amino acids in food.

### 2.8. Flavor Analysis of the Fermentation Liquid

Flavor analysis of the fermentation liquid was conducted according to the standards and technologies of the National Institutes of Health using the WILEY database [21].

### 2.9. Determination of TVB-N and Heavy Metal Levels

Volatile salt-based nitrogen analysis was conducted according to the GB 5009.228-2016 National Standard for Food Safety Determination of volatile salt-based nitrogen in food. Total arsenic analysis was conducted according to the GB 5009.11-2014 National Standard for Food Safety Determination of total arsenic and inorganic arsenic in food. Mercury analysis was conducted according to the GB 5009.17-2014 National Standard for Food Safety Determination of total mercury and organic mercury in food. Cadmium analysis was conducted according to the GB5009.15-2014 National Standard for Food Safety Determination of cadmium in food. Lead analysis was conducted according to the GB 5009.12-2017 National Standard for Food Safety Determination of lead in food.

### 2.10. Statistical Analysis

Data were analyzed using the SPSS software (version 21.0); the values were expressed as the mean ± standard deviation, and the means were used in Duncan’s test to compare. Statistical significance was set at *p* < 0.05. Origin 2018 software was used to summarize the experimental data and construct the images.

## 3. Results and Discussion

### 3.1. Best Conditions of Fermentation

**Figure 1:**
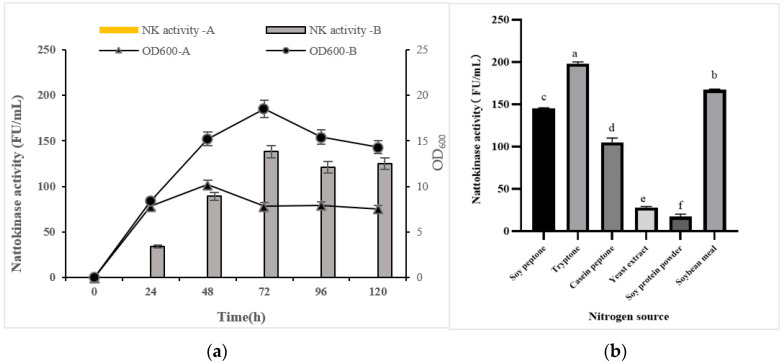
Best conditions of the fermentation for *PSP2*. (**a**) Growth and enzyme production in culture medium using oyster peptide as the sole nitrogen source (A) or in LB medium (B). (**b**) The enzyme production of oyster protein hydrolysate coexisting with additional nitrogen sources (*p* < 0.05). Different lowercase letters in the figure indicate significant differences between values.

First, *PSP2* was inoculated into the culture medium using oyster protein hydrolysate as the sole nitrogen source, and the enzymatic activity of nattokinase and optical density at 600 nm (OD_600_) were determined. As shown in Figure 1a, OD_600_ increased rapidly during the first 24 h of fermentation, indicating that the bacteria proliferated rapidly. However, the rapid growth of *PSP2* was only observed for a short period when oyster protein hydrolysate was used as the sole nitrogen source, indicating that the strain could not effectively utilize oyster protein hydrolysate to produce nattokinase. In the LB medium, *PSP2* showed improved growth and produced nattokinase. Since *PSP2* did not produce nattokinase in a medium that only uses oyster protein hydrolysate as a nitrogen source, additional nitrogen sources are added to promote the production of nattokinase. Based on the growth characteristics of *B. subtilis*, soybean peptone, pancreatic peptone, casein peptone, yeast extract, soybean protein powder, and soybean meal powder were optimized as exogenous nitrogen sources. As shown in Figure 1b, a medium containing 1.0% oyster protein hydrolysate combined with 1.0% exogenous nitrogen promoted the growth and metabolism of *PSP2* and the production of Nattokinase. The nitrogen sources for the maximum enzyme activity of nattokinase produced by *PSP2* were tryptone > soybean meal > soybean peptone > casein peptone > yeast extract > soybean protein powder. These results revealed that tryptone promoted rapid growth and increased enzyme production by the *PSP2* strain.

**Figure 2:**
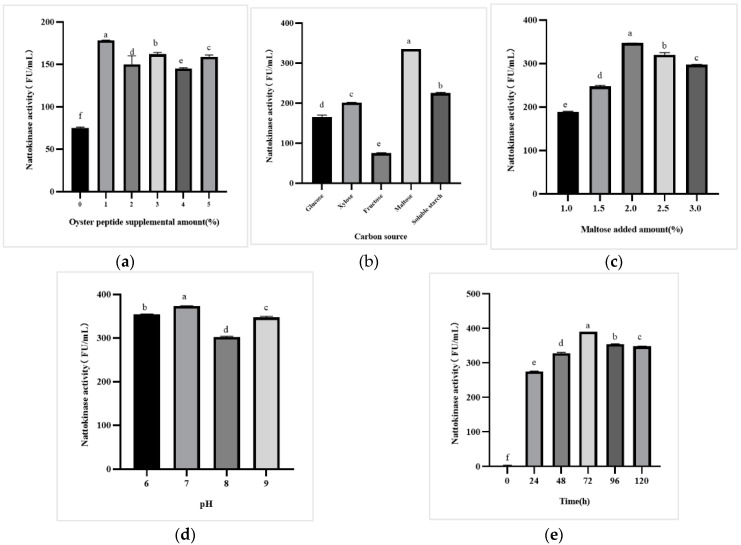
Effects of fermentation conditions on the production of nattokinase. (**a**) Nattokinase activity of *PSP2* in media with different oyster protein hydrolysate additions. (**b**) Nattokinase activity of *PSP2* in media with different carbon sources. (**c**) Nattokinase activity of *PSP2* in media with different maltose additions. (**d**) Nattokinase activity of *PSP2* in media with different initial pH. (**e**) Effect of fermentation time on the production of nattokinase by *PSP2* (*p* < 0.05). Different lowercase letters in the figure indicate significant differences between values.

We found when the oyster protein hydrolysate content was 0% and peptone content was 1.0%, low nattokinase activity was observed in the products after 72 h of fermentation (Figure 2a). However, when the dosage of oyster protein hydrolysate was increased from 1.0 to 5.0%, the enzyme activity of the products also increased. Nattokinase activity was 178.25 ± 3.84 FU/mL after 72 h of fermentation with 1.0% oyster protein hydrolysate, which was slightly better than that under other conditions. This indicates that the addition of oyster protein hydrolysate can increase the level of nattokinase produced by *PSP2*. Therefore, the 1.0% oyster protein hydrolysate combined with 1.0% tryptone was selected for subsequent experimental optimization. After determining the types of exogenous nitrogen sources and the amount of oyster protein hydrolysate, the effects of different carbon sources on the production of nattokinase from *PSP2* were determined to further improve nattokinase activity. Based on previous studies, glucose, xylose, fructose, maltose, and soluble starch were selected for further investigation. As shown in Figure 2b, the five carbon sources promoted enzyme production in the following order: maltose > soluble starch > xylose > glucose > fructose. The best carbon source was found to be maltose, which is consistent with the results of Pagnoncelli et al., who also found that maltose was the optimal carbon source. The optimized medium showed a high nattokinase activity of 1300 U/mL, which was six times higher than the original medium [22,23]. Following this, the amount of maltose was determined and the experimental results are shown in Figure 2c. Nattokinase activity in the products reached its maximum when the maltose content was 2.0%. When the concentration of the carbon source was low, the nutrients were insufficient, bacterial growth was slow, and the enzyme production efficiency was low. However, when the concentration of the carbon source was high, it easily caused aging and autolysis of bacteria [24]. Based on these results, the initial pH conditions for fermentation were optimized. The experimental results are shown in Figure 2d. There was a slight change in nattokinase activity between the different pH values selected, and 7.0 was chosen as the initial pH of fermentation. To determine the best conditions for the fermentation, the fermentation time was selected according to the law of enzyme production. Figure 2e shows that the maximum enzyme activity of the products was 390.23 ± 10.24 FU/mL after a fermentation period of 72 h.

Based on these results, optimal fermentation conditions were determined. In the medium containing 1.0% tryptone, 1.0% oyster protein hydrolysate, 2.0% maltose, and 0.5% sodium chloride, the initial fermentation pH was 7.0, and the maximum enzyme activity was 390.23 ± 10.24 FU/mL after 72 h of fermentation. Suwanmanon et al. reported enzymatic activity of 130.9 FU/mL under optimized fermentation conditions [25]. Zhang Q increased the activity of the nattokinase enzyme from 143.89 to 151.05 FU/mL after two-bacteria fermentation [26]. These results indicate that the level of nattokinase activity in this study is higher than that in previous studies.

### 3.2. Analysis of the Fermentation Liquid Components

The molecular composition, protein molecular weight, and amino acid composition of the medium before and after fermentation were analyzed. The product compositions are listed in Table 1. After fermentation, the solid content in the supernatant decreased from 4.82 to 3.81%, and the total sugar increased from 3.73 to 8.92%. The proportion of protein in the products before and after fermentation was 70–80%. Compared to the levels of amino acids at 0 h, the levels of all kinds of amino acids in the 72-h fermentation liquid decreased to different degrees. There are three main reasons for the decrease in various amino acid levels: (1) Glu, Arg, Lys, Tyr, and other amino acids under the action of amino acid decarboxylase generate different kinds of biological amines, such as putrescine, in the fermentation process; (2) amino acids, such as Arg and Lys, are involved in the Strecker degradation reaction in the middle stage of the Maillard reaction, and alcohol–aldehyde condensation, aldehyde–ammonia polymerization, and cyclization reactions in the late stage of the Maillard reaction to generate melanoids and other substances; and (3) the consumption rate of some amino acids, such as *PSP2*, which cannot be synthesized by itself and is needed in the process of growth and reproduction, was higher than the degradation rate of the protease substrate.

As shown in Table 2, the molecular weight of the oyster fermentation liquid at 72 h was higher than that at 0 h. This is because, in addition to producing nattokinase during fermentation, *PSP2* uses oyster protein hydrolysate and other ingredients in the culture to enhance its own growth and metabolism to produce complex proteins.

### 3.3. Flavor Analysis of the Fermentation Liquid

Enzymolysis is the main technology used in oyster product development; however, after enzymolysis treatment, the unpleasant oyster odor worsens, seriously affecting consumer acceptance. Fishy taste is a widespread issue, and aquatic products generally taste earthy, musty, fishy, and rancid. The components of fishy substances in aquatic products are complex, and their formation mechanisms can be analyzed based on the following aspects: oxidative degradation of lipids, decomposition of aromatic precursor substances, catalytic decomposition of enzymes, and accumulation of microbial metabolites [27]. The levels of volatile substances in the fermentation products at 0 and 72 h were determined and compared with those in the database (Table 3).

There were 14 flavor components in the 0 h fermentation liquid and 26 flavor components in the 72-h fermentation liquid, indicating that fermentation enhanced the flavor and produced many kinds of pyrazines. These were mainly obtained via the participation of different amino acids in the Maillard reaction, which is consistent with the results of the amino acid analysis mentioned above. The proportion of bad flavor components in the 0 h fermentation liquid was 15.15% and the proportion of components contributing to a pleasant smell was 26.69%. In the 72-h oyster fermentation liquid, the proportion of bad flavor components was 12.24%, and the proportion of components contributing to a pleasant smell was 69.08%. After *PSP2* fermentation, the levels of isovaleraldehyde, hexanal, heptaldehyde, nonaldehyde, and other major unpleasant odor components were greatly reduced. Substances such as pyrazines, which contribute to pleasant smells, increased significantly, and the oyster peptide flavor was also improved [28].

### 3.4. Determination of TVB-N and Heavy Metal Levels

TVB-N refers to the decomposition of proteins caused by enzymes and bacteria during food spoilage to produce basic nitrogenous substances, such as ammonia and amines. This is an important factor for assessing fermentation processes. These substances are volatile, and the higher their content, the more the amino acids are destroyed, especially methionine and tyrosine. As a result, the nutritional value of products is also greatly affected. As shown in Table 4, TVB-N levels in the fermented products at 0 and 72 h were 71.8 ± 1.2 and 68.4 ± 0.4 mg/kg, respectively. TVB-N levels decreased, indicating that *PSP2* did not produce volatile salt nitrogen easily compared to the fermentation process of wild bacteria. Generally, the levels of arsenic, mercury, lead, and cadmium in aquatic products should be monitored. Based on the toxicity intensity, the inorganic arsenic content in the total arsenic and methyl mercury content in the total mercury should be considered. As shown in Table 4, the total arsenic, total mercury, lead, and cadmium levels in the fermented oyster protein hydrolysate after fermentation decreased and were all within the limited range [29]. In 1980, some researchers found that the cell wall of *B. subtilis* has carboxyl and other functional groups that can be used as deposition sites for metal ions and to exert an adsorptive effect [30]. Later, many researchers found that *Bacillus* spp. can adsorb lead, cadmium, and other heavy metals. This may explain why the content of heavy metal ions in this study decreased after fermentation.

### 3.5. ACE Inhibitory Activity

**Figure 3:**
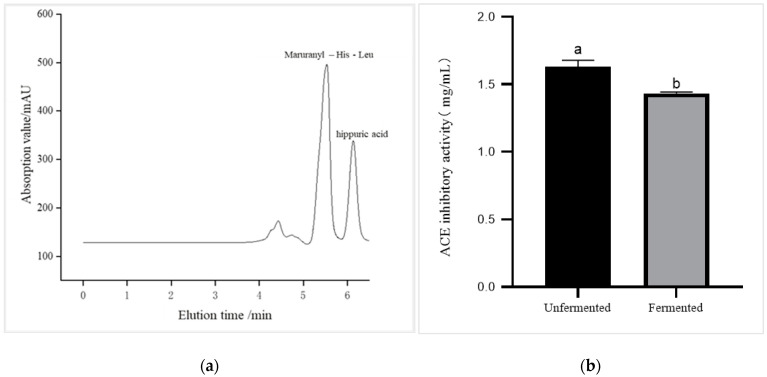
(**a**) HPLC diagram for assay of ACE inhibition activity. (**b**) Results of ACE inhibition activity in oyster fermentation liquid (*p* < 0.05). Different lowercase letters in the figure indicate significant differences between values.

As shown in Figure 3b, the ACE inhibitory activity level of the peptides was 1.629 ± 0.041 mg/mL and 1.433 ± 0.008 mg/mL before and after oyster protein hydrolysate fermentation, respectively. After synergistic fermentation of oyster protein hydrolysate and *PSP2*, the ACE inhibitory activity increased slightly. Wang et al. obtained an oyster ACE inhibitory peptide with an IC_50_ value of 66 μmol/L in vitro [15]. Hua et al. obtained an NPARTCR peptide with high ACE inhibition potential through isolation, and further digestion with proline-specific endopeptidase produced an ACE inhibitory activity IC_50_ of 77.0 μmol/L [31]. Zhang et al. analyzed the structure–activity relationship and structural characteristics of ACE-inhibitory peptides and selected proline-specific endopeptidases combined with chymotrypsin to prepare oyster protein hydrolysate with high ACE inhibitory activity levels. The IC_50_ of the products was 0.128 mg/mL [14].

The low inhibition level of ACE in the products obtained in this study may be due to the low specificity of the neutral protease and papain used in the enzymatic hydrolysis process and nattokinase produced in the fermentation process. Peptide hydrolysates produced via non-specific enzymatic hydrolysis may contain more peptides with no ACE-inhibitory activity. In addition, oyster protein hydrolysate is also used as a nitrogen source to supplement bacterial growth and enzyme production in the process of *PSP2* fermentation.

## 4. Conclusions

In this study, we obtained oyster protein hydrolysate fermentation products with nattokinase activity and both antithrombotic and hypotensive properties, which provides an effective fermentation technology for the development of marine functional foods. It also has the potential for application in the treatment of chronic metabolic diseases in humans. Moreover, the flavor of the product was improved, and some toxic and harmful substances, such as heavy metals, were partially removed after fermentation. We aim to further investigate effective industrial fermentation approaches to reduce or eliminate unpleasant odors and to develop marine functional foods with antithrombotic and hypotensive properties as well as high sensory acceptance.

## Figures and Tables

**Table 1:** Changes of total sugar, total protein, and amino acid contents in oyster fermentation liquid.

	Oyster Fermentation Liquid (0 h)	Oyster Fermentation Liquid (72 h)
Solid content (%)	4.82 ± 0.23 ^a^	3.81 ± 0.18 ^b^
Total sugar (%)	3.73 ± 0.54 ^b^	8.92 ± 1.37 ^a^
Total protein (%)	69.29 ± 1.25 ^b^	78.21 ± 0.74 ^a^
Asp (%)	10.14 ± 0.47 ^a^	9.86 ± 0.25 ^a^
Thr (%)	5.28 ± 0.23 ^a^	5.03 ± 0.12 ^a^
Ser (%)	5.03 ± 0.15 ^a^	4.08 ± 0.22 ^b^
Glu (%)	14.95 ± 0.76 ^a^	14.24 ± 0.42 ^a^
Pro (%)	4.54 ± 0.47 ^a^	3.72 ± 0.22 ^b^
Gly (%)	6.53 ± 0.81 ^a^	6.45 ± 0.13 ^a^
Ala (%)	10.52 ± 1.12 ^a^	10.42 ± 0.94 ^a^
Val (%)	5.47 ± 0.14 ^a^	4.63 ± 0.29 ^b^
Met (%)	2.25 ± 0.11 ^a^	2.09 ± 0.45 ^a^
Ile (%)	4.51 ± 0.12 ^a^	3.63 ± 0.33 ^b^
Leu (%)	7.47 ± 0.47 ^a^	7.03 ± 0.65 ^a^
Tyr (%)	3.88 ± 0.05 ^a^	3.08 ± 0.03 ^b^
Phe (%)	4.46 ± 0.12 ^a^	3.66 ± 0.22 ^b^
Lys (%)	10.20 ± 0.98 ^a^	10.14 ± 0.74 ^a^
His (%)	1.52 ± 0.03 ^a^	1.48 ± 0.05 ^a^
Arg (%)	3.05 ± 0.08 ^a^	2.99 ± 0.14 ^a^

Different letters indicate significant differences between the two sets of values.

**Table 2:** Proportion changes of oyster protein hydrolysate with different molecular weights in oyster fermentation liquid.

Samples	Molecular Weight Distribution	
Oyster fermentation liquid (0 h)	Molecular weight range (kDa)	Proportion (%)
	3~50	94.72 ± 1.25
	50~150	3.53 ± 0.54
	150~14,800	1.75 ± 0.70
Oyster fermentation liquid (72 h)	60~85	42.08 ± 1.56
	85~310	54.30 ± 1.18
	More than 310	3.62 ± 0.35

**Table 3:** Proportion of volatile flavor substances in fermented products (%).

Volatile Substance	Smell	Oyster Protein Hydrolysate (Unfermented)	Oyster Protein Hydrolysate (Fermented)
Isovaleraldehyde	Stench	4.07 ± 0.48	
Hexanal	Clams taste	3.15 ± 0.63	
Heptylaldehyde	Clams taste stench	5.10 ± 1.14	
Nonylaldehyde	Clams taste	2.83 ± 0.53	
Benzaldehyde	Contribute to pleasant smells	9.21 ± 1.25	14.16 ± 1.18
2-Furyl alcohol	Contribute to pleasant smells	7.48 ± 0.68	
3,4-Dimethylbenzaldehyde	Contribute to pleasant smells	2.54 ± 0.82	
1,2, 3-Trimethylbenzene	Contribute to pleasant smells	0.94 ± 0.07	
1,3, 5-Trimethylbenzene	Contribute to pleasant smells	1.70 ± 0.20	
Silane	Contribute to pleasant smells	7.36 ± 0.94	
Furfurylalcohol	Contribute to pleasant smells	2.51 ± 0.73	5.12 ± 0.04
Cyanobenzene	Contribute to pleasant smells	10.40 ± 0.91	
O-methyl benzonitrile	Contribute to pleasant smells	29.02 ± 1.44	
Methoxy-phenyl-oxime	Stench		6.51 ± 1.26
2, 4-Di-tert-butylphenol	Anxious burnt smell		5.73 ± 0.97
Dimethyl disulfide	Contribute to pleasant smells		2.01 ± 0.45
2-Heptyl ketone	Contribute to pleasant smells		7.18 ± 0.38
6-Methyl-2-heptanone	Contribute to pleasant smells		2.57 ± 0.18
2-Methylpyrazine	Contribute to pleasant smells		0.31 ± 0.22
2, 5-Dimethylpyrazine	Contribute to pleasant smells		35.31 ± 1.44
2-Ethyl-5-methylpyrazine	Contribute to pleasant smells		2.07 ± 1.01
2,3, 5-Trimethylpyrazine	Contribute to pleasant smells		1.16 ± 0.08
2-Decyl ketone	Contribute to pleasant smells		0.68 ± 0.36
2-Ethylhexanol	Contribute to pleasant smells		0.94 ± 0.53
Benzene ethanol	Contribute to pleasant smells		6.79 ± 1.12
Phenylacetonitrile	Contribute to pleasant smells		5.90 ± 0.90

**Table 4:** TVB-N and heavy metal content in oyster protein hydrolysate.

Contents	Oyster Protein Hydrolysate (Unfermented)	Oyster Protein Hydrolysate (Fermented)
TVB-N (mg/100 g)	7.18 ± 0.12 ^a^	6.84 ± 0.04 ^b^
Total arsenic (mg/kg)	0.100 ± 0.003 ^a^	0.087 ± 0.012 ^a^
Total mercury (mg/kg)	<0.003	<0.003
Lead (mg/kg)	<0.020	<0.020
Cadmium (mg/kg)	0.056 ± 0.005 ^a^	0.039 ± 0.001 ^b^

Different letters indicate significant differences between the two sets of values.

## Data Availability

Data are contained within the article.
